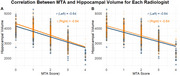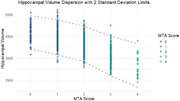# Analysis of the relationship between volumetry and visual classification of hippocampal atrophy on magnetic ressonance image

**DOI:** 10.1002/alz70856_106082

**Published:** 2026-01-11

**Authors:** Fabricio Nery Garrafiel, Ricardo Benardi Soder, Ricardo Pessini Paganin, Maria Rosa Alves da Silva, Andrei Bieger, Vitor Verlindo Vidaletti, Cristiano Aguzzoli, Lucas Porcello Schilling

**Affiliations:** ^1^ Pontifícia Universidade Católica do Rio Grande do Sul (PUCRS), Porto Alegre, Rio Grande do Sul, Brazil; ^2^ Instituto do Cérebro do Rio Grande do Sul, Porto Alegre, RS, Brazil; ^3^ Pontifical Catholic University of Rio Grande do Sul, PORTO ALEGRE, RIO GRANDE DO SUL, Brazil; ^4^ Universidade Federal do Rio Grande do Sul, Porto Alegre, Rio Grande do Sul, Brazil; ^5^ Neurology Department, São Lucas Hospital of PUCRS, Porto Alegre, Rio Grande do Sul, Brazil; ^6^ Brain Institute of Rio Grande do Sul (InsCer), Porto Alegre, Rio Grande do Sul, Brazil; ^7^ Global Brain Health Institute (GBHI), San Francisco, CA, USA; ^8^ School of Medicine, Pontifícia Universidade Católica do Rio Grande do Sul (PUCRS), Porto Alegre, Rio Grande do Sul, Brazil

## Abstract

**Background:**

Magnetic resonance imaging (MRI) is a tool used in the evaluation of patients with cognitive deficits, capable of identifying characteristic changes of neurodegenerative processes, such as hippocampal atrophy. The most common assessment methods include scoring systems using the Fazekas and MTA scales. The present study aims to evaluate the relationship between the classification data from the MTA scale, obtained through the analysis of MRI images by two experienced neuroradiologists, and the data obtained from hippocampal volumetry of the same image sample.

**Method:**

677 MRI images were collected from the Alzheimer's Disease Neuroimaging Initiative (ADNI) database and the hippocampal volume quantification values were extracted using Freesurfer. The degree of atrophy, according to the MTA scale, on both sides of the hippocampus were classified by two independent specialized radiologists.

**Result:**

The correlation between MTA classifications was 0.85, and a negative correlation (‐0.64) was found between volumetry and atrophy grade (fgure 1). Despite these results correlating the MTA grade with automatic volumetry, approximately 10% of the individuals were classified as outliers, being determined to be outside 2 standard deviations from the mean volume of each atrophy grade (Figure 2).

**Conclusion:**

The results showed a strong correlation between the MTA scale and volumetry, as well as a consistent correlation between the radiologists' MTA classification. Albeit we identified 10% of individuals classified as outliers, highlighting the existence of specific cases that should be better investigated in order to understand the efficiency of the qualitative method and the accuracy of its classifications.